# A novel doxorubicin/CTLA-4 blocker co-loaded drug delivery system improves efficacy and safety in antitumor therapy

**DOI:** 10.1038/s41419-024-06776-6

**Published:** 2024-06-01

**Authors:** Wenli Yang, Qinghui Sun, Xiaodian Zhang, Liping Zheng, Xiaomei Yang, Na He, Yanyang Pang, Xi Wang, Zhiheng Lai, Wuping Zheng, Shaoping Zheng, Wu Wang

**Affiliations:** 1 Public Research Center, Hainan Medical University, Haikou, China; 2 Department of Anatomy, Zunyi Medical University, Zunyi, China; 3 Department of Ultrasound, Union Hospital, Tongji Medical College, Huazhong University of Science and Technology, Wuhan, China; 4 Department of Breast and Thyroid Surgery, The Second Affiliated Hospital of Hainan Medical University, Haikou, China; 5 School of Tropical Medicine, Hainan MedicalUniversity, Haikou, China; 6 Hainan Cancer Medical Center of The First Affiliated Hospital, Hainan Engineering Research Center for Biological Sample Resources of Major Diseases, Hainan Medical University, Haikou, China; 7 Department of Breast Surgery, The First Affiliated Hospital of Hainan Medical University, Haikou, China; 8 Guangxi Key Laboratory of Nanobody Research/Guangxi Nanobody Engineering Research Center, Guangxi Medical University, Nanning, Guangxi China; 9 School of Traditional Chinese Medicine, Hainan Medical University, Haikou, China; 10 Department of Anesthesiology, Haikou Third People’s Hospital, Haikou, China; 11 Department of Anorectal, Hainan Province Hospital of Traditional Chinese Medicine, Haikou, China

**Keywords:** Cancer microenvironment, Drug delivery

## Abstract

Doxorubicin’s antitumor effectiveness may be constrained with ineffective tumor penetration, systemic adverse effects, as well as drug resistance. The co-loading of immune checkpoint inhibitors and doxorubicin into liposomes can produce synergistic benefits and address problems, including quick drug clearance, toxicity, and low drug penetration efficiency. In our previous study, we modified a nanobody targeting CTLA-4 onto liposomes (LPS-Nb36) to be an extremely potent CTLA-4 signal blocker which improve the CD8^+^ T-cell activity against tumors under physiological conditions. In this study, we designed a drug delivery system (LPS-RGD-Nb36-DOX) based on LPS-Nb36 that realized the doxorubicin and anti-CTLA-4 Nb co-loaded and RGD modification, and was applied to antitumor therapy. We tested whether LPS-RGD-Nb36-DOX could targets the tumor by in vivo animal photography, and more importantly, promote cytotoxic T cells proliferation, pro-inflammatory cytokine production, and cytotoxicity. Our findings demonstrated that the combination of activated CD8^+^ T cells with doxorubicin/anti-CTLA-4 Nb co-loaded liposomes can effectively eradicate tumor cells both in vivo and in vitro. This combination therapy is anticipated to have synergistic antitumor effects. More importantly, it has the potential to reduce the dose of chemotherapeutic drugs and improve safety.

## Introduction

Doxorubicin (DOX) represents one of the most potent antineoplastic medications as well as the most widely active compound among similar drugs. It can be used to treat solid tumors and hematological malignancies, including breast lung, bile duct, prostate, uterus, ovary, stomach and liver tumors, etc. [1–3]. The antitumor mechanism of DOX lies in its capacity to be incorporated into the DNA helix as well as bind covalently, to proteins that participate in DNA replication as well as transcription, which results in inhibition of DNA and protein synthesis in tumor cells [4]. More and more studies have shown that the application of liposomes to be drug delivery vectors may enhance the concentration of DOX in tumor sites and the internalization of specific cells and achieve rapid release in focal areas. Meanwhile, to reduce the DOX-induced cardiotoxicity and toxicity within various organs like the liver or kidney, liposomal delivery systems were developed to passively target DOX to tumor [5, 6]. There are currently several clinical trials underway for liposomal DOX, and several products such as Doxil [7] (FDA, breast carcinoma, oophoroma, myeloma, and Kaposi’s sarcoma), Myocet [8] (Europe, metastatic breast carcinoma) have been approved for antitumor therapy in different countries. Even with this progress, there are still issues with liposomal DOX that prevent it from being used in clinical settings. Adverse effects in several organs, such as the liver, kidneys, and heart, in addition to dose-related side effects [9]. Chemotherapy induces immune system suppressive effects that raise the risk of tumor recurrence and spread, as well as the eventual development of drug resistance. Chemotherapy alone is unable to completely and permanently destroy the tumor microenvironment [10]. In clinical practice, the standard of care for treating cancer has changed beyond the empirical application of cytotoxic therapy to customized therapy. Targeted therapy or adoptive cellular immunotherapy, as well as the use of immune checkpoint blockers, make the treatment of tumor patients more precise and sustainable. Additional studies show that tumor immunotherapy significantly affects the prognoses for multiple cancers, as well as chemotherapy combined with immunotherapy has advantages in inhibiting tumor persistence and disrupting tumor microenvironment [11].

Between immune cells in the tumor microenvironment and tumor cells during the tumor formation process, there is a complicated regulation mechanism. These mechanisms include positive regulation, which activates the immune system to attack the tumor, and negative regulation, which weakens the activity of immune cells, which together determine the development or suppression of the tumor. Among these, immune checkpoints signal pathways (CTLA-4/B7,PD-1/PDL-1,TIM-3, etc.) play an essential function [12]. Cytotoxic T-lymphocyte-associated antigen-4 (CTLA-4) blockers have drawn attention to be one of the early immune checkpoint signal processes within the tumor’s microenvironment because they significantly reduced tumor activity and were used in clinical settings [13]. CTLA-4 has become a protein receptor mainly produced on the surface of triggered CD4^+^ as well as CD8^+^ T cells (or intracellular proteins in non-activated cells) as a homolog of CD28. While the particular binding of CTLA-4 to B7 (mainly CD80 or CD86) is enhanced, that leads to competitive inhibition of CD28/B7 co-stimulatory signals, thus inhibiting T-cell growth and triggering, resulting in decreased secretion for cytokines such as IL-2, IL-4, IFN-γ, as well as decreased expression for IL-2 receptor, which creates the condition for tumor cells to escape immune surveillance and makes the rapid development of tumors [14, 15]. Currently, the FDA has approved various CTLA-4 monoclonal antibodies (ipilimumab, tremelimumab) for use in clinical studies as well as for certain types of malignancies, including monotherapy and combination therapy [16]. Combination therapy with CTLA-4 blockers and DOX or other chemotherapeutic drugs is thought to be a potential method for combating chemotherapy’s immune system-inhibiting effects while lowering the dosage of chemotherapeutic medications to increase safety [17, 18]. The difficulties at the moment are as follows: (1) The high molecular weight of CTLA-4 mAb (160–170 kDa) prevents its penetration into the tumor microenvironment as well as maintains its high concentration inside the tumor. (2) The chemotherapeutic drugs and CTLA-4 blockers entering tumors lack targeting. (3) The side effects of treatment. (4) The complex production process of CTLA-4 mAb bring high production cost [19]. Therefore, it is urgent to develop new strategies for DOX combined with CTLA-4 blockers with high efficiency, as well as low cost for attaining more effective antitumor therapy results.

**Fig. 1:**
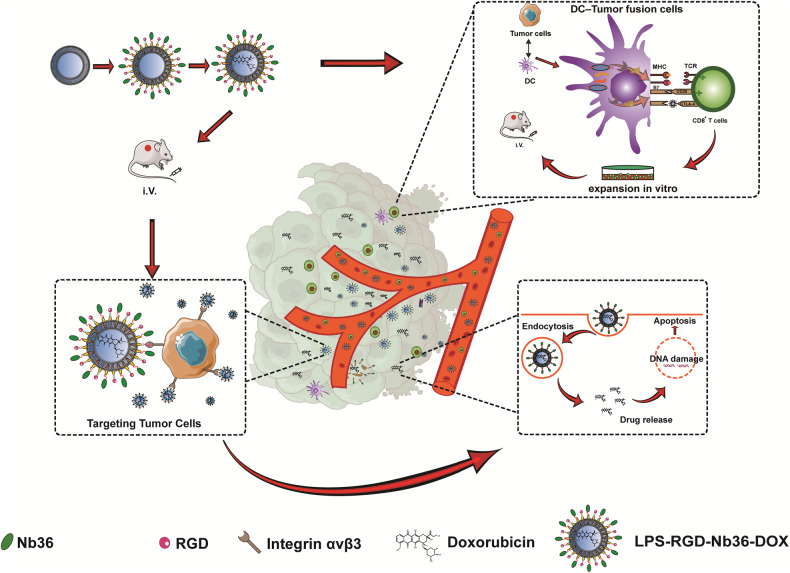
Therapy strategy with FC + LPS-RGD-Nb36-DOX treatment. DC-tumor fusion vaccines were first generated. Then, in addition to delivering DOX that targets tumor cells, the LPS-RGD-Nb36-DOX complex eliminated immunosuppression via blocking CTLA-4-mediated negative co-stimulation in the CD8^+^ T cells stimulated by DC/tumor fusion vaccine.

In our previous study, a nanobody (Nb) targeting CTLA-4 (Nb36) was examined in a prior work using phage display technology [20], and it was shown that it binds effectively to CTLA-4 epitopes upon activating T cells within vitro [21]. Nb, the smallest antigen-binding fragment generated from camelid animals, has a greater capacity for antigen identification and binding than mAb, making it potentially very useful within the detection as well as management for malignant tumors [22, 23]. With these inherent advantages, Nb36 can be utilized to create an extremely potent CTLA-4 signal blocker (LPS-Nb36) when modified onto liposomes which enhances its antigen-binding ability. Our data show that Nb36 was more suitable for enrichment on liposomes than mAb, thus improving the CD8^+^ T-cell antitumor capacity in vitro when combined with with DC/tumor fusion vaccines [24]. Therefore, based on its outstanding compatibility and toxicity identification, we postulated that LPS-Nb36 might be able for being immediately utilized within the creation of in vivo medicinal systems for drug delivery. In this study, we constructed a drug delivery system (LPS-RGD-Nb36-DOX)based on LPS-Nb36 that realized the doxorubicin and anti-CTLA-4 Nb co-loaded and was applied to antitumor therapy in vivo. RGD ligand, which allows RGD to specifically identify integrin receptors on the surface for tumor cells, was added to the liposomes as a supplementary modification [25]. Figure 1 shows how MAL-PEG2000-DSPE linked Nb36 to the surface of liposomes, which enhances its antigen-binding ability due to the high concentration while preventing the CTLA-4/B7 signaling process for T cells. After identifying and binding to CTLA-4 positive target cells, Nb36 was shed from the liposomes because of its greater binding force with CTLA-4 epitope. Subsequently, the liposomes might additionally internalize into the tumor cells through RGD focusing. Furthermore, these liposomes endocytosed into tumor cells and accumulate in the tumor tissue after administration, followed by inhibition of tumor cells DNA synthesis and strong induction apoptosis through the release of DOX.

The creation of potential liposomes altered with anti-CTLA-4 Nb and RGD to function as a fresh CTLA-4 molecule blocker with tumor targeting is first covered in this paper. When these liposomes are loaded with DOX, a co-delivery system is achieved. We investigated the anticancer activity as well as the mechanism utilizing DC/tumor fusion vaccines-stimulated tumor-specific CD8^+^ T cells in combination with LPS-RGD-Nb36-DOX. As a combination therapy strategy, these effector T cells can be continuously stimulated by LPS-RGD-Nb36-DOX before adoption and after entering the tumor. For another, as a co-loaded drug delivery system, LPS-RGD-Nb36-DOX can simultaneously deliver DOX and Nb36 into tumors. We anticipate that the combination of this chemotherapy and immunotherapy approach will result in a lower dosage of DOX, increased antitumor efficaciousness, and enhanced safety.

## Materials and methods

### Animals and cells

The American Type Culture Collection provided human gastric cancer cells A-427 as well as human hepatocellular carcinoma cells Hep3B. A-427 was grown on RPMI-1640 medium (Gibco, USA), while Hep3B was grown on MEM (Gibco, USA). Both were kept at 37 °C with 5% CO_2_ and contained 10% fetal bovine serum (Hyclone, USA), 100 U/ml of penicillin, and 100 mg/ml of streptomycin. Female NOD/SCID mice of SPF grade had been purchased from Zhuhai Baitry Tong Biomedical Technology Co., LTD. (Zhuhai, China) and maintained in an SPF environment. The mice ranged in age from 4 to 6 weeks. All protocols were approved by the Hainan Medical University’s animal ethics committee. All animal experiments were carried out in compliance with the National Institutes of Health’s guide for the care and use of laboratory animals and the ARRIVE recommendations.

### LPS-RGD-Nb36-DOX generation

The thin-film hydration approach was utilized to prepare maleimide-modified unloaded liposomes [24, 26]. The production and purity of Nb36 were performed by *Escherichia coli* WK6 cells and detailed steps have been reported in our previous study [20].

Through the use of the post-insertion technique, Nbs and RGD have been attached to the liposome surface [27]. In a flask, Nb36, RGD tripeptide (treated with dithiothreitol), as well as MAL-PEG2000-DSPE have been incorporated at a molar ratio for 1:10, sealed with nitrogen, and incubated for 24 h at 4 °C. Get LPS-RGD-Nb36 utilized HEPES liquid dialysis after removing the free small molecules.

The ammonium sulfate gradient method was employed for the DOX loading of LPS-RGD-Nb36 [28]. In an eggplant bottle, combine LPS-RGD-Nb36, HSPC, and CHOL Lipid Reserve Fluid. Add the necessary amount of chloroform, dissolve, and stir. Rotate the vial under vacuum to evaporate the chloroform, dry it up, and create a lipid film. (NH_4_)_2_SO_4_-HEPES buffer was added for shock, then the liposome solution was ultrasound to transparency by ultrasonic crushing apparatus, and finally transferred to a dialysis bag with 10 kDa molecular weight interception for dialysis to prepare liposomes with ammonium sulfate gradient. According to the ratio of lipid: doxorubicin =10:1(w/w), LPS-RGD-Nb36-DOX was obtained.

A dynamic laser particle size analyzer (NanoBrook 90plus PALS, Brookhaven, USA) was used to determine the size, distribution range, as well as zeta potential of the produced liposomes, and a transmission electron microscopy (TEM, Jem-F200, JEOL, Japan) was used to capture the liposomes’ morphology. The encapsulation rate and release rate of liposome were measured by conventional methods, and the drug loading capacity was calculated.

### Specific binding of LPS-RGD-Nb36-DOX

In our previous study, Nb36 was screened by using phage display technology, which involves the construction of anti-CTLA-4 Nbs libraries, screening for Nbs, and the purification of Nbs in Escherichia coli host [20]. In Library construction, a His-tag and HA-tag were added to the sequence of Nbs and cloned into the phagemid vector. Since Nb36 was expressed, His-tag, an anti-His-tag mAb (Abcam, UK, Cat No. ab213204) could be utilized as a secondary antibody. After the co-incubation of LPS-RGD-Nb36-DOX with activated CD8^+^ T cells, flow cytometry was performed to analyze their fluorescent signal. In another part, these cells incubated with LPS-RGD-Nb36-DOX were reincubated with anti-CTLA-4 mAb (Abcam, USA), and CTLA-4 expression was examined to reflect the specific binding of LPS-RGD-Nb36-DOX to CD8^+^ T cells.

### Fusion of tumor cells and DCs

The procedures used to generate CD8^+^ T cells and DCs are based on earlier research [24]. Hep3B and A-427 tumor cells were obtained during the logarithmic development stage, exposed to 30 GyX of radiation to render them inactive, and then combined with PKH26 fluorescent dye (Sigma-Aldrich, USA). Following a 2:1 co-cultivation ratio with tumor cells, DCs were combined with CFSE fluorescent dye (Sigma-Aldrich, USA) and put into a 50-ml centrifuge tube. PEG (Sigma, USA) was then added, followed by it had been water-bathed at 37 °C for five minutes. After that, the diluted collagen I (Sigma, USA) was incorporated, and it soaked for 30 min under a water bath at 37 °C.

The fusion cells were examined using fluorescence microscopy (Nikon ECLIPSE 80i, Nikon, Japan) following PBS washing. Following seven days of incubation, these fusion cells were harvested, and flow cytometry (Backman CytoFlex S) was used to measure the expression levels of MHC II, CD80, and CD86 in order to evaluate the DCs maturation. Data analysis was done using FlowJo version 10.0. Using the previously mentioned fluorescence microscope, the fusion cells’ fluorescence level had been recorded.

### Cell proliferation assay

For the goal of evaluating whether LPS-RGD-Nb36-DOX may increase effector T-cell growth, specific categories of effector T cells had been pre-marked by 7-AAD as well as then co-cultivated with DC/Hep3B fusion cells or DC/A-427 fusion cells at a 10:1 ratio for 5 days.

In total, 5 μg/ml of Nb36, LPS-RGD-Nb36 (an equimolar amount of 5 μg/ml Nb), LPS-RGD-Nb36-DOX (an equimolar amount of 5 μg/ml Nb), LPS, or an equimolar amount of anti-CTLA-4 mAb(Abcam, UK, Cat No. ab210384) have been employed to culture these cells. The aforementioned flow cytometry has been employed to evaluate the 7-AAD fluorescence on effector T cells.

### Cytotoxicity assay

For cytotoxicity assay, distinct sets for activated CD8^+^ T cells had been co-cultured with AnexinV-APC-labeled target cells (Hep3B and A-427) at a ratio of 1:1, 5:1, 10:1, or 20:1 for 6 h.

The LPS-DOX (or LPS-DOX+Nb36, LPS-DOX+anti-CTLA-4 mAb), LPS, LPS-RGD-Nb36 as well as LPS-RGD-Nb36-DOX have been included correspondingly combined DC/tumor fusion vaccines triggered CD8^+^ T cells at the specific DOX concentration. Then harvest these target cells and dyed with 7-AAD (Keygen Biotech, China). Next, the ratios of AnexinV-APC^+^7-AAD^+^ cells all of the groups had been analyze with flow cytometry as a % for the particular lysis.

In addition, each group’s effecter T cells had been co-cultured at a 20:1 ratio with target cells and treated with anti-CD107a antibodies (Biolegend, China, Cat No.328605). The intracellular IFN-γ tests were carried out using a Cytofix/Cytoperm™ Fixation/Permeabilization Kit from BD Biosciences in the United States. The aforementioned flow cytometry was used to assess the ratios of positive effector T cells.

### ELISA and ELISPOT assays

Separate cohorts of CD8^+^ T cells stimulated by the DC/tumor fusion vaccination were co-cultured 1:1 with either A-427 or Hep3B cells. Using commercially available ELISA kits, the levels of IL-2, TNF-α, IFN-γ, and IL-10 in the supernatants were assessed (BD Biosciences, USA). The liposomes and antibodies were introduced in the aforementioned dosages, respectively. In the meantime, an ELISPOT kit (Mabtech, Sweden) was used to assess the percentage of these cells that secreted IFN-γ specifically. Subsequently, the immunological complex specific to IFN-γ was subjected to streptavidin-AP processing, and it was observed in the BCIP/NBT substrate solution. To find the amount of spots, a Cellular Technology Limited ImmunoSpot S6 Ultimate-V analyzer has been applied.

### In vivo animal photography

Hep3B as well as A-427 cells (5 × 10^5^ cells/mouse) had been through the skin injected into NOD/SCID mice under their left armpit. Every three days, the tumor volume was measured using the formula 0.5ab^2^ (a = greatest diameter; b = perpendicular diameter). Mice were randomly assigned to a total of six categories (5 mice per category) and given the appropriate therapy (IR-780 at a dosage of 0.1 mg/ml) when their tumors grew to a size of about 100 mm^3^. Since IR-780’s absorption wavelength is ~780 nm, it was chosen as the image tracker in vivo because it enables real-time in vivo distribution monitoring. The in vivo imaging system (IVIS Lumina XR, PerkinElmer, USA) calculated the fluorescence imaging seen in the tumor at particular intervals after treatment. The organs and tumors from the animals have been collected after 12 h for additional ex vivo photography.

### In vitro cellular uptake

In all, 24-well plates were seeded with 5 × 10^4^ Hep3B as well as A-427 cells during the logarithmic development stage. For 2, 4, and 6 h, the LPS-RGD-Nb36-DOX was incorporated at a DOX dosage of 10 μg/ml. Following DAPI staining, the nuclei were cleaned with PBS. Finally, using the aforementioned flow cytometry, the cells’ fluorescence intensity was calculated and quantified.

### Toxicity evaluation of LPS-DOX

To determine the safe dose of LPS-DOX, NOD/SCID mice were tail vein injected with different doses of LPS-DOX. Every day, changes in the mice’s weight as well as biological behavior were observed. The mice were killed 48 h after the treatment began, and the heart, liver, spleen, lung, kidney, as well as serum had been taken out. Samples of tissue had been fixed in 10% neutral formalin, embedded in paraffin, sectioned into 4-µm-thick sections, and stained with hematoxylin and eosin (HE). The mice were given serum samples to be tested biochemically using an AU5800 Clinical Chemistry Analyzer (Beckman Coulter, Inc.). Mice’s liver and heart function were evaluated using the enzymes alanine aminotransferase (ALT), aspartate transaminase (AST), creatine kinase (CK), CK-MB, as well as lactate dehydrogenase (LDH-L).

### Modeling of xenograft tumors and in vivo therapy

The referenced subcutaneous transplanted tumor model in the above is Hep3B and A-427 mice. To further validate the effect of LPS-RGD-Nb36-DOX combined with activated CD8^+^ T cell therapy on primary tumors, we constructed a PDX (patient-derived tumor xenograft) mouse models of primary liver cancer. Patients diagnosed with hepatocellular carcinoma (HCC) have been chosen to receive surgically excised primary tumor tissue (all participants gave informed agreement). The research received approval from Hainan Medical University’s institutional ethics committee. Next, the collected primary tumor tissue was cut into 3–4 mm slices and inoculated subcutaneously into the left armpit for 6-week-old male NOD/SCID mice after anesthesia with ether. Tumor-bearing mice were sacrificed, and their tumors were removed. The tumors were then cut up in a sterile environment and injected into NOD/SCID mice in the manner previously mentioned in order to facilitate ongoing transplantation. Once each mouse’s tumor grew around 100 mm^3^, the animals were randomized separated into groups and given intravenous treatment with CD8^+^ T cells that had been activated by the DC/tumor fusion vaccination and had been pre-incubated with liposomes or antibodies. Meanwhile, mice in each group were injected with corresponding liposomes and antibodies. The tumor volume was recorded every 3 days. Following the final recording, the mice had been euthanized,, their tumors removed and retained for further assays.

### Immunohistology

For the purpose to examine the tumor’s effector T-cell infiltration and tumor cell viability. Next, these tumor sections were subjected to immuno-staining with anti-Ki-67 mAb (Biolegend, China, Cat No. 328605), and the results were analyzed. As directed by the provider, the immunofluorescence technique (TUNEL, FITC, in situ Cell Death Detection Kit, Roche, Switzerland) has been applied to ascertain the apoptosis of tumor cells. Photographs have been taken using a fluorescence microscope.

### In vivo survival and antitumor efficacy of CD8^+^ T cells

On days 7, 14, and 28 following treatment, three modeled mice had to be killed in both groups. After obtaining the spleen, peripheral blood, as well as tumor tissues, the tissues had been cut into tiny fragments and then homogenized. Using flow cytometry, the ratio of CD8 expression was discovered. Lastly, on day 28, ELISA was used to determine the serum levels of IL-2 and IFN-γ.

### Statistical analysis

Software called GraphPad Prism 9.0.0 has been employed to statistically analyze the data. Students’ *t* tests as well as a calculation of variance (ANOVA) were used to assess the data. Animal survival curves varied across categories, and the Kaplan–Meier analysis (log-rank test) was used to depict these curves. The definition of statistical significance was a *P* value of less than 0.05. The means ± standard deviation (SD) were used to display all the data.

## Results

### Characterization and specific binding of LPS-RGD-Nb36-DOX

LPS-RGD-Nb36-DOX’s drug loading capacity was 6.21%, and its encapsulation rate was 92.88% (Supplementary Fig. S1). LPS-RGD-Nb36-DOX’s size, dispersion, and form were examined using a transmission electron microscope (TEM, JEM-F200, JEOL, JAPAN). Our data demonstrate that LPS-RGD-Nb36-DOX had a nearly monodisperse distribution and a more uniform, spherical or elliptically spherical size. The diameters of the LPS-RGD-Nb36-DOX, LPS-RGD-Nb36, LPS-DOX, and LPS particles depicted in Supplementary Fig. S2A were all less than the value of about 110 nm obtained in Supplementary Fig. S2C from the dynamic laser particle size analyzer. This could be explained by the fact that liposomes measured by the particle size analyzer were in an aqueous state, whilst those seen via transmission electron microscopy were dry. As a result, the liposomes with the hydrated membrane have a larger particle size. In the meantime, the low PDI of these liposomes (Supplementary Table S1) suggests a very homogeneous dispersion of them. Furthermore, the significant stability for LPS-RGD-Nb36-DOX is confirmed by our ZETA potential data (Supplementary Fig. S2B).

Next, we looked into the possibility that LPS-RGD-Nb36-DOX might attach to stimulated CD8^+^ T cells specifically. Because Nb36 has been generated with a His-tag, a fluorescent signal may be produced by using an anti-His-tag monoclonal antibody. Our findings demonstrate that the His-tag expression effectiveness approached 39.5% (Supplementary Fig. S2D) while LPS-RGD-Nb36-DOX had been incubated with these T cells, confirming the liposomes’ considerable ability to attach to the CTLA-4 molecule on the surface of activating CD8^+^ T cells. In a reverse verification, the binding effectiveness of CTLA-4 in LPS-RGD-Nb36-DOX incubated CD8^+^ T cells was examined using CTLA-4 mAb. The findings demonstrated that the CTLA-4 antigen on T cells actually binds specifically to LPS-RGD-Nb36-DOX.

### LPS‑RGD-Nb36-DOX enhance activated CD8^+^ T cells proliferation and activation in vitro

**Fig. 2:**
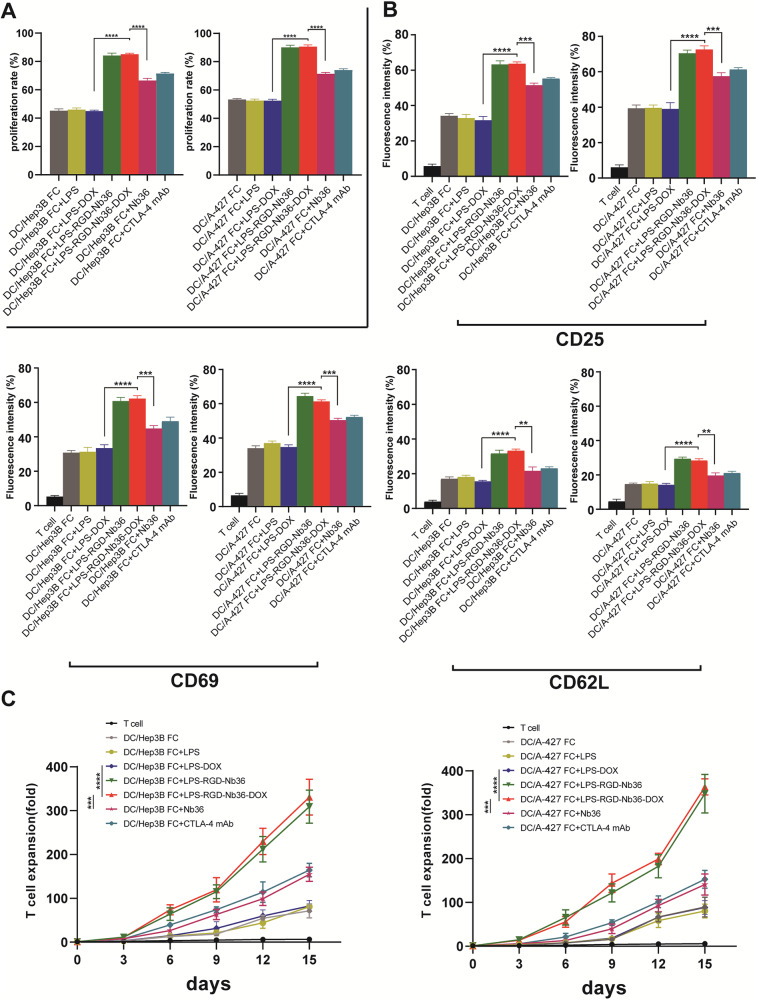
LPS-RGD-Nb36-DOX enhances activation and proliferation of CD8^+^ T cells in FC + LPS-RGD-Nb36-DOX treatment. **A** The CD8^+^ T cells were stained with CFSE and co-culture with Hep3B or A-427 cells, respectively, with LPS-RGD-Nb36-DOX, LPS-RGD-Nb36, Nb36, or anti-CTLA-4 mAb for 5 days and analyzed by flow cytometry for the activation. *n* = 3, ****P < 0.0001. **B** The CD8^+^ T cells above were stained with CFSE-conjugated anti-CD25 or anti-CD69 or CD62L mAb and analyzed by flow cytometry for activation. *n* = 3, **P < 0.01, ***P < 0.001, ****P < 0.0001. **C** The expansion fold of The CD8^+^ Tin each group co-cultured with Hep3B or A^-^427 cells. *n* = 3, ***P < 0.001, ****P < 0.0001.

The DC/tumor fusion vaccine-induced autologous CD8^+^ T-cell stimulation as well as multiplication can be further advanced by LPS-RGD-Nb36-DOX. We first successfully finished the preparation of DC/tumor fusion Cells. The DC/tumor fusion cells that we produced had strong expression of maturity-associated markers (MHC II, CD80, and CD86) as well as both types of fluorescence (Supplementary Fig. S3A, B). Our observations revealed a significant increase in CD25, CD69, and CD62L expression in LPS-RGD-Nb36-DOX as well as DC/tumor fusion vaccine-activated CD8^+^ T cells (*P* < 0.001) (Fig. 2B). In the meantime, Fig. 2A, C shows that LPS-RGD-Nb36-DOX greatly increased the effector T cells growing (*P* < 0.001). In conclusion, the DC/tumor fusion vaccine stimulated CD8^+^ T-cell growth and activity can be enhanced by LPS-RGD-Nb36-DOX. Furthermore, LPS-RGD-Nb36-DOX is a more effective T-cell CTLA-4/B7 signal inhibitor than Nb36 and anti-CTLA-4 mAb in this particular therapy.

### LPS‑RGD-Nb36-DOX enhance the secretion of inflammatory cytokines of activated CD8^+^ T cells

**Fig. 3:**
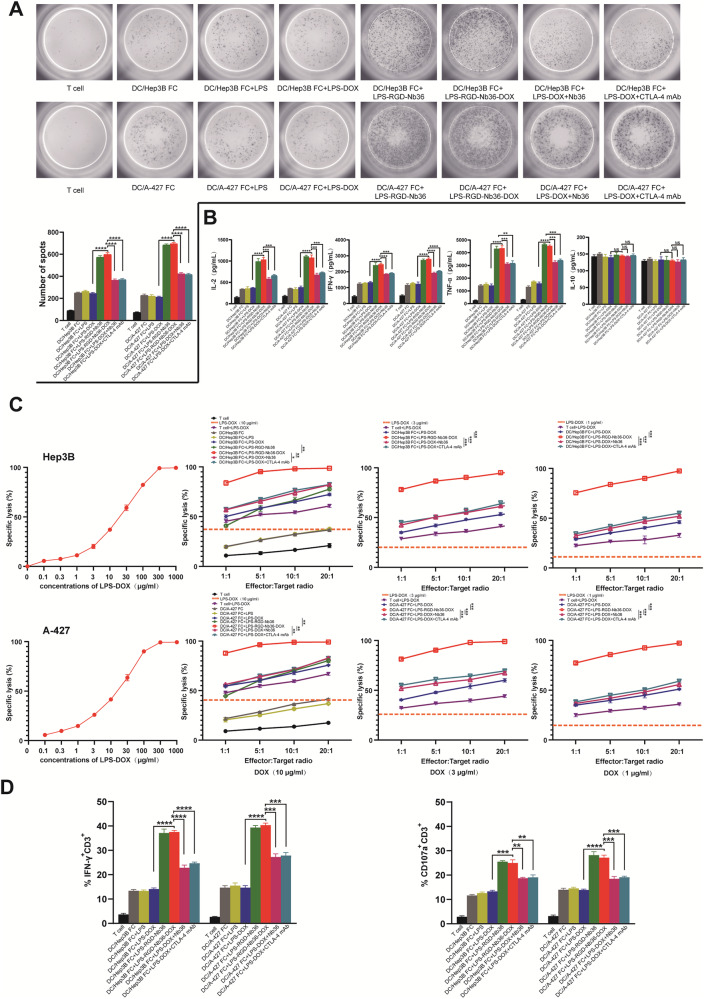
LPS-RGD-Nb36-DOX enhances inflammatory cytokine secretion and cytotoxicity of CD8^+^ T cells in DC/tumor fusion + LPS-Nb36 treatment. The sorted human CD8^+^ T cells were cultured and activated by corresponding DC/tumor fusion vaccine alone or in the presence of LPS-RGD-Nb36-DOX, LPS-RGD-Nb36, Nb36 or anti-CTLA-4 mAb, then stimulated with the same number of irradiated Hep3B or A-427 cells for 24 h in vitro. CD8^+^ T cells cultured without DC/tumor fusion vaccine as the T cells group. **A** The secretion of IFN-γ in the activated CD8^+^ T cells of different groups against the stimulations of corresponding tumor cells was detected by ELISPOT assay to indicate the significant involvement of LPS-RGD-Nb36-DOX and LPS-RGD-Nb36 with the increased amount of IFN-γ-producing CD8^+^ T cells inducing by DC/tumor-FC vaccines. Representative images of ELISPOT plate readout analyzing the frequency of IFN-γ-secreted CD8^+^ T cells are shown histograms represent data of the triplicates for 3 × 10^5^ cells from three independent ELISPOT assays, and shown as bars of means + SD. ****P < 0.0001. **B** After co-culture with effector cells and target cells, supernatants were separated and analyzed for secretion of IL-2, IFN-γ, TNF-α, and IL-10 and measured by the ELISA kits. Results indicated the increased production of the above three cytokines from the tumor cell-reactive CD8^+^ T cells mediated by liposomal drugs. Bar graphs show mean of cytokine concentration + SD. *n* = 3, **P < 0.01, ***P < 0.001, ****P < 0.0001. **C** The CD8^+^ T cells were co-incubated with AnexinV-APC^-^prestained Hep3B or A-427 cells with DC/tumor fusion vaccine at E/T ratios 1:1, 5:1, 10:1, or 20:1, with different doses of liposomal drugs for 6 h. 7-AAD was used for lysed cell staining. The ratios of AnexinV-APC^+^ 7-AAD^+^ cells were measured by flow cytometry^.^ *n* = 3, ^*^*P < 0.01, ***P < 0.001. **D** After co-culturing with target cells at a 20:1 ratio and a 10 μg/ml liposomal drug dose for 6 h, the IFN-γ (intracellular) and CD107a. *n* = 3, **P < 0.01, ***P < 0.001, ****P < 0.0001.

To investigate the production for cytokines within effector T cells in order to verify their cytotoxicity mechanism, we conducted ELISA to evaluate the supernatant for these T cells after exposure to tumor antigen. The results we obtained demonstrated that the effector T cells in LPS-RGD-Nb36-DOX treatment exposure with irradiated Hep3B and A-427 cells lead to an improved discharge for inflammatory factors including IL-2, IFN-γ as well as TNF-α in contrast to the control category (*P* < 0.001) (Fig. 3B). There was an increase in cytokine release levels compared to the FC + LPS-DOX+Nb36 groups. It is important to note that the categories’ levels of the IL-10 did not differ statistically significantly (*P* > 0.05).

Our ELISPOT data elucidated that the IFN-γ secreting spot forming within the DC/tumor fusion+ LPS-RGD-Nb36-DOX categories were elevated compared with those within the other control group after being challenged with Hep3B and A-427 cells (Fig. 3A). Thus, our data confirm that LPS-DOX+Nb36 enhances the growth and responsiveness for activated CD8^+^ T cells to pro-inflammatory factors, which would lead to enhance local inflammatory response and cytotoxic on tumor cells.

### Activated CD8^+^ T cells combine LPS-RGD-Nb36-DOX treatment effectively eliminate tumor cells in vitro

As demonstrated by our research, when activated CD8^+^ T cells are combined with LPS-RGD-Nb36-DOX therapy, a greater proportion of matching tumor cells may be eliminated. We evaluated the ability of each treatment approach to kill tumor cells at various DOX dosages by utilizing the LPS-DOX dose gradient in relation to target cell death. Our data show that in addition to the apoptosis directly caused by DOX uptake into target cells, such CD8^+^ T cells can also demonstrate substantial cytotoxicity that results in a higher effectiveness killing proportion (Fig. 3C).

On the other hand, their killing effect on the tumor cells at various DOX dose gradients had been likewise noticeably larger compared to those of the FC + LPS-DOX, FC + LPS-DOX+Nb36, FC + LPS-DOX+anti-CTLA-4 mAb and FC + LPS-RGD-Nb36 groups. more evidence that the combination of LPS-RGD-Nb36-DOX and activated CD8^+^ T cells strategy formed a notable synergistic effect which is better than that of single action. It is noteworthy that, in contrast to other control groups, the FC + LPS-RGD-Nb36-DOX group’s ability to kill target cells does not actually diminish considerably as the DOX dose drops. This indicates that the DOX/CTLA-4 blocker co-loaded strategy’s antitumor therapy may lower the dosage of chemotherapy medications and increase patient safety.

In order to assess the cytotoxicity of such CD8^+^ T cells in relation to the corresponding tumor cells, we used flow cytometry to identify intracellular IFN-γ as well as the mobilization for CD107a, which is indicative of the cytotoxic phenotype, throughout the membrane for these effector T cells. The findings showed that FC + LPS-RGD-Nb36-DOX groups had increased intracellular IFN-γ as well as CD107a expression (Fig. 3D), which is in line with the assay’s results for vitro cytotoxicity.

### In vitro cellular uptake and in vivo biodistribution of LPS‑RGD-Nb36-DOX

**Fig. 4:**
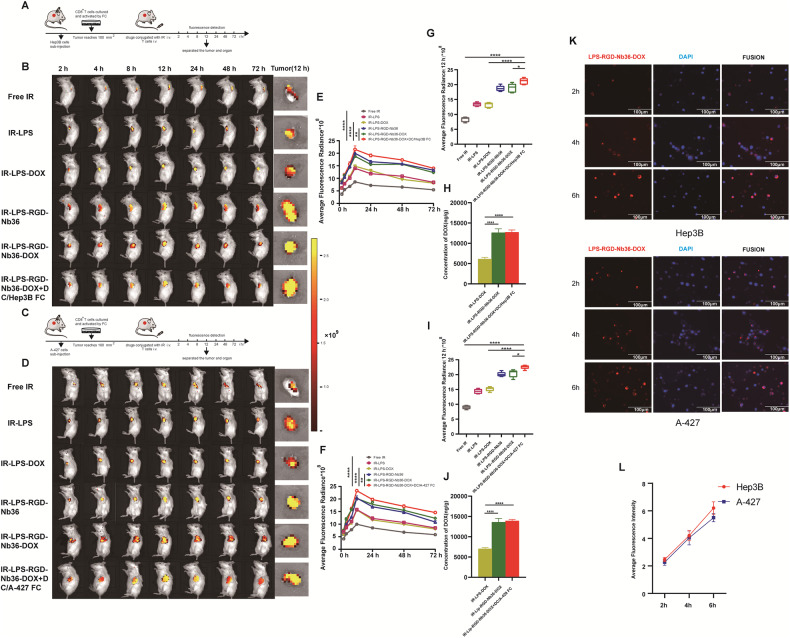
In vitro cellular uptake and in vivo biodistribution of LPS-RGD-Nb36-DOX. **A**–**D** Fluorescence signal distribution of Hep3B or A-427 cell tumor-bearing model at 2, 4, 8, 12, 24, 48, and 72 h post-injection of Free IR, IR-LPS, IR-LPS-DOX, IR-LPS-RGD-Nb36, IR-LPS-RGD-Nb36-DOX, and IR-LPS-RGD-Nb36-DOX + FC-activated CD8^+^ T cells. **E**, **F** Average fluorescence radiance of tumor site at each time point. *n* = 3. **P < 0.01, ****P < 0.0001. **G**, **I** Average fluorescence radiance of exfoliated tumor. *n* = 3. *P < 0.05, ****P < 0.0001. **H**, **J** High-Performance Liquid Chromatography–Mass Spectrometry (HPLC-MS) determined the concentration of doxorubicin in the tumor. *n* = 3. ****P < 0.0001. **K**, **L** Representative fluorescence microscopy images of Hep3B or A-427 cells after incubation with LPS-RGD-Nb36-DOX for 2 h, 4 h, and 6 h, respectively. *n* = 3.

We calculated the target cells binding characteristics of LPS-RGD-Nb36-DOX, which are essential to its efficiency and specificity. To assess the effectiveness of RGD-mediated cellular absorption of LPS-RGD-Nb36-DOX, fluorescence microscopy was used. Our research indicates that LPS-RGD-Nb36-DOX can precisely attach to the target cell’s membrane surface protein, as demonstrated by the way they exhibit greater red fluorescence intensity and strength with time (Fig. 4K, L). The significant tumor cells binding capacity of LPS-RGD-NB36-DOX suggests that it may have great potential for delivering drugs into tumors when used in vivo, therefore, the in vivo imaging system had been conducted to confirm the shipping actions for LPS‑RGD-Nb36-DOX in both Hep3B and A-427 bearing mice model, respectively. In these two tumor-bearing mice. Mice in all groups containing liposomal formulations had more tumor accumulation than those in the free IR-780 group, the EPR effect may be responsible for this. The fluorescence level of the tumor site within the free IR-780 category decays throughout a 24-h period, while a significant fluorescent signal is retained in those groups containing liposomal formulations (Fig. 4A–G, I). Tumors in these liposomal formulation-treated mice fluoresced more intensely compared to normal organs, indicating that the tumor cells were able to be selectively absorbed and that DOX released quickly after internalization of the cells (Supplementary Fig. S4). HPLC-MS was performed to detect DOX concentration in the exfoliated tumors, as well as the outcomes corresponded with the above (Fig. 4H, J). After receiving therapy with FC + LPS-RGD-Nb36-DOX, mice showed more fluorescence signals at tumor locations when compared to other groups. It might be due to that the modification of RGD on the surface of liposomes, as well as the signal enhancement caused by some activated CD8^+^ T cells that bind the LPS‑RGD-Nb36-DOX to target the tumor site.

### Activated CD8^+^ T cells combine LPS-RGD-Nb36-DOX treatment leads to a higher antitumor effect in tumor-bearing mice

**Fig. 5:**
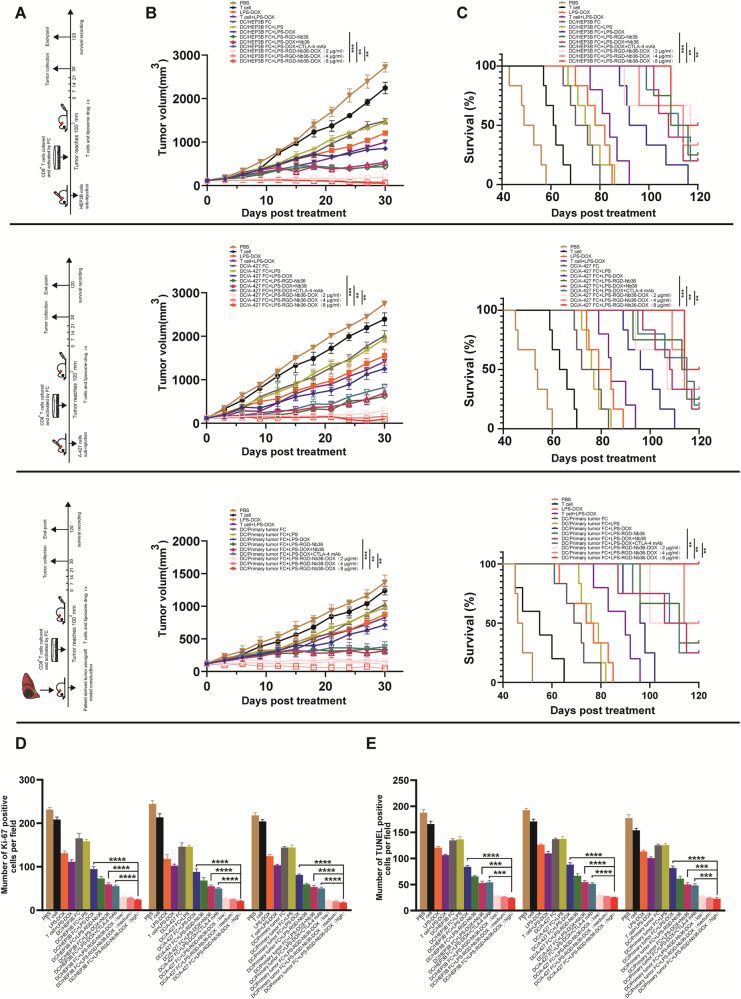
FC + LPS-RGD-Nb36-DOX treatment lead to a higher antitumor function in xenograft mice. **A**, **B** The tumor size was measured every 3 days. *n* = 5, **P < 0.01, ***P < 0.001. **C** Kaplan–Meier survival curve of each group. *n* = 5, **P < 0.01, ***P < 0.001. **D** Ki-67 detection by immunohistochemical stain. *n* = 5, ****P < 0.0001. **E** The number of apoptosis cells detection by TUNEL. *n* = 5, ***P < 0.001, ****P < 0.0001.

To establish the safe dose, we administered several doses of LPS-DOX into healthy mice prior to in vivo. Our data showed that when LPS-DOX was administered at a dose lower than 8 mg/kg, no abnormalities were observed in mouse behavior, no damage was shown in major organs, and there had been not a substantial variation within ALT, AST, CK, CK-MB, and LDH-L levels, therefore, 8 mg/kg was confirmed as the highest safe dose for DOX (Supplementary Fig. S5A, B). With the construction of PDX mouse model completed (Supplementary Fig. S6A, B), our data confirmed that liposome as a vector can load DOX and transport it to the tumor site to induce apoptosis of tumor cells. Meanwhile, The CD8^+^ T cells in response to the activation of the DC/tumor fusion vaccine and CTLA-4 blocker activation enhanced survival and cytotoxicity. This combination therapy retarded tumor growth and strengthened survival of xenografted mice. Our data confirmed that Liposome as a vector can load DOX and transport it to the tumor site trigger apoptosis for tumor cells. Meanwhile, The CD8^+^ T cells within reaction to the stimulation by the DC/tumor fusion vaccine as well as CTLA-4 blocker activation enhanced survival and cytotoxicity. This combination therapy retarded tumor development as well as bolstered survival for xenografted mice (Fig. 5A, B). In addition, our results demonstrate that the combination of activated CD8^+^ T cells and LPS-RGD-Nb36-DOX therapy can sustain a comparatively potent antitumor effect even after DOX dosage is reduced to 2 mg/kg, which is anticipated to enhance the safety of antitumor therapy using this approach.

The antitumor mechanism was validated by measuring Ki-67 and apoptosis in the tumor using immunohistochemistry or immunofluorescence staining after activated CD8^+^ T cells combined LPS-RGD-Nb36-DOX treatment. The percentage of Ki-67-positive cells was lower in the FC + LPS-RGD-Nb36-DOX group than in the control, according to our findings. This outcome corresponded with the TUNEL assay’s strong apoptosis detection (Fig. 5D, E). All things considered, our findings show that adoptive activated CD8^+^ T cells, in response to the DC/tumor fusion vaccine and LPS-RGD-Nb36-DOX, limit the growth of implanted tumors in mice by preventing tumor cell division and increasing tumor cell death.

### Extended antigen-dependent longevity of CD8^+^ T cells in vivo

**Fig. 6:**
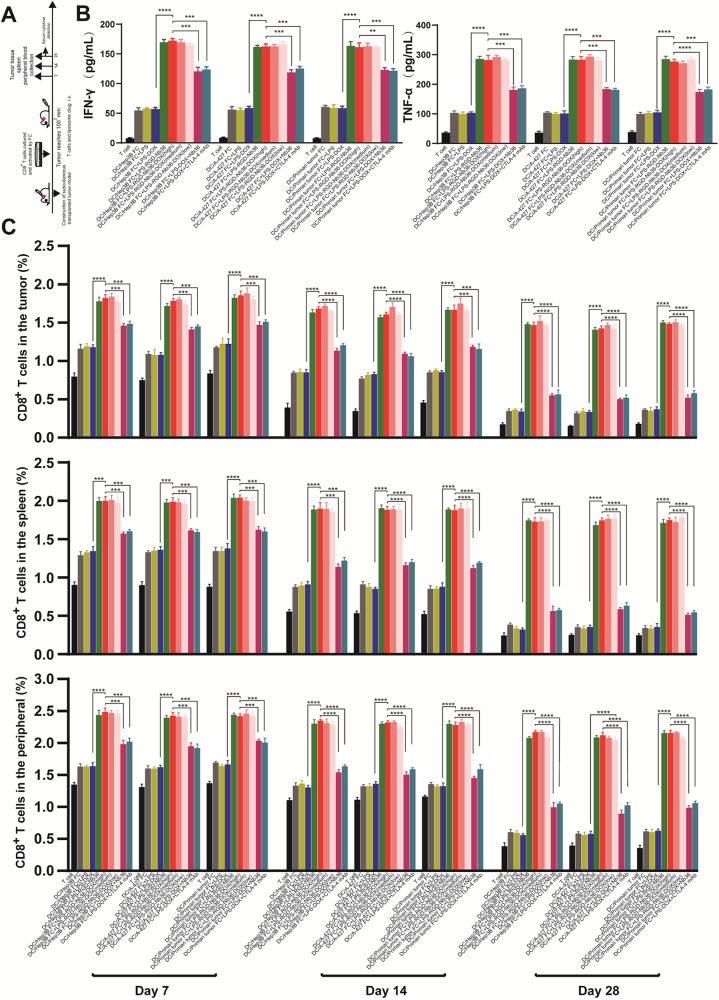
Long-term antigen-dependent persistence of CD8^+^ T cells in FC + LPS-RGD-Nb36-DOX treatment in vivo. **A**, **B** Human IFN-γ and TNF-α levels in tumor-bearing mice serum were detected by ELISA after adoptively transferring with effector T cells in each group on day 28. *n* = 3, **P < 0.01, ***P < 0.001, ****P < 0.0001. **C** The percentage of human CD8^+^ T cells in a tumor, spleen, and blood of tumor-bearing mice that were adoptively transferred with effector T cells in each group were measured by flow cytometry on days 7, 14, and 28. *n* = 3, *** *P* < 0.001, ****P < 0.0001.

Following a complete course of therapy, the adoptive CD8^+^ T-cell persistence within mice in all groups was assessed in vivo on days 7, 14, as well as 28 (Fig. 6A). Various tissues were collected from these mice, including tumors, blood and spleens, for human CD8 evaluation. Our flow cytometry data showed that CD8^+^ T cells activated through FC + LPS‑RGD-Nb36-DOX treatment persisted throughout the experiment (Fig. 6C). These CD8^+^ T cells continued to exhibit persistent anticancer activity on day 28, despite a small drop in the tumor, blood, and spleen. In addition, we ran a cytokine analysis on the mice’s serum that was collected on day 28. The results of the experiment demonstrated that, in comparison to the other groups, the mice in the FC + LPS-RGD-Nb36-DOX category had considerably greater amounts of human IFN-γ as well as TNF-α (Fig. 6B). These findings imply that these CD8^+^ T cells may be able to prolong their survival in mice’s bodies and consistently raise the local inflammation, which would result in long-term tumor suppression.

## Discussion

Over the past 20 years, two types of antitumor therapy, particularly the use of immune checkpoint inhibitors to boost naturally occurring anticancer activity and conventional chemotherapy medications, have been considered to be completely separate routes in cancer treatment [29, 30]. In addition to undergoing significant technological advancements as separate paths for research as well as development, these two therapies have also seen the introduction of stand-in medications, especially immune checkpoint inhibitors based on tumor immunity, which have become a hot spot in tumor therapy in the last decade.

Despite several CTLA-4 monoclonal antibody drugs have been recommended by the FDA for clinical trials in some cancers, they still face numerous challenges in terms of application such as complex structure, high immunogenicity, non-specifically binds to normal tissues and high production cost [31]. Nb, one of the single-domain antibodies, were derived from camelidae or some shark species and is being considered a better substitute for mAbs. As an alternative to mAbs, one of the strategies is the utilization of nanobodies (Nb) as an immune checkpoint inhibitor [22, 32]. In addition to serve as an antitumor antibody drug, Nb has also been reported to have the potential to be used in the construction of trispecific and CAR T cells [33, 34]. The particular inherent advantages give Nb great potential value in the fields of malignant tumor treatment and diagnosis. We tried using LPS-Nb36 to stimulate activated CD8^+^ T cells in our earlier study [24], and we saw some degree of increased cell activity, which improved effector cell cytotoxicity and survival. Still, this single adoptive immunotherapy strategy clearly has the potential to improve. From the current perspective, a single therapy has obvious advantages and disadvantages, resulting in clinical limitations. Tumor immunotherapy with many modalities is increasingly gaining traction in studies and development. It is now commonly acknowledged that certain chemotherapy and immunotherapy can work in concert to enhance therapeutic results [35, 36].

Liposomes are microencapsulated spherical carrier preparations made of phospholipids and cholesterol. Liposomes can achieve prolonged circulation in vivo following PEG modification, which makes them appropriate for the creation of drug delivery systems that include different medications for use in antitumor therapy, such as paclitaxel and doxorubicin [37]. The advantages of liposomes as drug delivery vector lie in their good biocompatibility, biodegradability, and low toxicity. Because of the tumor tissue’s increased permeability and retention (EPG) impact, it can cause specific aggregation of loaded macromolecular drugs or nanoparticles at the tumor site, thus forming a certain passive targeting effect [38]. Because amphiphilic medications are hydrophilic when they are ionized and are unable to penetrate the lipid bilayer, they are lipid soluble when they are not ionic, therefore, the loading of drugs in liposomes is often achieved by chemical gradients [39]. Consequently, in order to manufacture drug-loaded liposomes and achieve a reasonably high encapsulation rate, we employed the ammonium sulfate gradient approach. However, the EPR effect of liposomes is not sufficient to increase the amount of drug reaching the focal area to a satisfactory concentration. More importantly, it is frequently challenging to disperse medications evenly throughout the tumor due to the tumor interstitial barrier, which is created by a variety of reasons, including disorderly proliferation of the tumor stroma, high hydraulic pressure of the tumor stroma, and abnormalities of neovasculogenesis [40]. Thus far, there has been a growing interest in active-targeted drug delivery systems. The smallest known integrin identification sequence capable of binding to the αvβ_3_ receptor is thought to be RGD tripeptide [41]. Numerous investigations have verified that RGD alteration on liposomes is a well-developed design scheme that can improve drug-loaded liposomes’ ability to target tumors and raise the concentration of the medication in tumors [42, 43]. Therefore, we designed the liposome modification of RGD to facilitate the movement of Nb36 and DOX into the tumor interior.

In addition, early research has shown that adoptive immunotherapy plus liposomal DOX can increase the overall anticancer effect [44]. Nevertheless, the problem is that these effector T cells are inhibited by the tumor microenvironment, and it is difficult to obtain lasting activity. Tumor-specific cytotoxic T lymphocytes, which are activated CD8^+^ T cells and are stimulated by DCs or cancer vaccines, are actually nevertheless far from ideal, particularly for within in vivo uses. Numerous variables impede the generation and stimulation of tumor-specific CTLs in vivo, but immunological checkpoint negative regulation is particularly significant. Several DC/tumor fusion vaccines developed in our earlier research [45, 46] indicated that effector T-cell survival in mice fell short of expectations and that they were unable to permanently impede tumor development and recurrence.

In this study, with the goal to improve CD8^+^ T cells growth and sustainability, LPS-RGD-Nb36-DOX simultaneously loaded DOX and Nb36, and used the Nb36 as an immune checkpoint blocker, which realized the antitumor strategy of chemotherapies combined with immunotherapy in vivo. Our data demonstrate that, following drug loading, LPS-RGD-Nb36-DOX exhibits homogeneous particle size as well as dispersion with no noticeable modifications in liposome characterization. Because of the enhanced tumor targeting following EPR effect and RGD modification, when LPS-RGD-Nb36-DOX was administered to tumor-bearing mice, the drug concentration rose in both the tumor region and the tumor cells. The capacity to specifically target tumors leads to more accurate tumor destruction by liberating DOX from LPS-RGD-Nb36-DOX. In addition to the direct pharmacological damage of tumor cells, on the other hand, the combined application of LPS-RGD-Nb36-DOX and activated CD8^+^ T cells activated the antitumor activity for these effector cells, further enhancing the cellular immunity attack on tumor cells. In vitro, LPS-RGD-Nb36-DOX exhibits a binding rate that appears to be comparable to that of Nb36 or anti-CTLA-4 mAb, and it can specifically bind to the CTLA-4 antigen on the surface of CD8^+^ T cells. When the CTLA-4/B7 axis was upregulated on the effector T cells, the developed LPS-RGD-Nb36-DOX was employed as an effective CTLA-4 blocker to lessen the detrimental effect. As anticipated, upon encountering tumor antigens, the tumor-specific CTLs in conjunction with LPS-RGD-Nb36-DOX were able to quickly blockade the CTLA-4/B7 axis, as seen by increased expression of CD25, CD69, and CD62L as well as increased cytokine secretion (TNF-α, IFN-γ, and IL-2). The results of our investigation demonstrate a significant synergistic effect between tumor-specific T cells and anti-CTLA-4 Nb/DOX co-loaded liposomes. When this medication stimulates effector T cells, they become more cytotoxic, have higher survival rates, and are better able to target tumors, which increases the rate at which tumor cells undergo apoptosis.

The clinical antitumor use of DOX is limited by DOX-induced cardiotoxicity and toxicity in the liver and kidneys. In order to lessen DOX’s negative adverse reactions, the clinical antitumor dose for DOX has to be strictly limited. Numerous initiatives were undertaken to lessen DOX’s negative impacts, including combined antioxidant and/or anti-apoptotic compounds, the design of efficacious delivery systems, and the synthesis of DOX analogs [47–49]. However, in clinical trials or animal models that are relevant to medicine, the majority of techniques have not been able to lessen the toxicity of DOX [50]. We tried to validate different doses of DOX in combination with activated CD8^+^ T cells against tumors. With this approach, a lower dose of DOX can still result in a high in vitro tumor cell death rate for the combo therapy. This synergistic effect of chemotherapy combined with immunotherapy may be involved to the mechanism by which inducing immunogenic cell death (ICD). Tumor cell debris may function as a “vaccine” in this process, stimulating the immune cells with comparatively small dosages of DOX, hence improving overall efficiency. In vivo experiments, To substantiate the LPS-RGD-Nb36-DOX+ activated CD8^+^ T cells treatment in attacking tumor cells within vivo, a mouse subcutaneous transplanted tumor model of liver cancer and lung cancer, as well as a PDX model about primary liver cancer, were constructed. Following toxicity testing, the safe range’s maximum therapeutic dose was found to be 8 mg/kg. Our data confirmed that this treatment strategy of LPS-RGD-Nb36-DOX in combination with activated CD8^+^ T cells delayed tumor development as well as enhanced the longevity for each of the models of mice. Remarkably, even with the DOX dose reduced to 2 mg/kg, the treatment group with LPS-RGD-Nb36-DOX+ activated CD8^+^ T cells remains the most successful. These outcomes further demonstrated that the DOX dosage can be greatly decreased by using chemotherapy in conjunction with immunotherapy to increase the safety of antitumor therapy.

Investigating how this treatment approach kills tumors showed a marked reduction among the proliferative (Ki-67) and apoptotic (TUNEL) properties of tumor cells within mice, a finding in line with the targeted destruction of DOX and effector T cells on tumor cells. We also examined the effector cells’ ability to survive in mice. Tumor metastasis and recurrence may be caused by the limitation of effector cells within an extremely invasive solid tumor microenvironment. The long-term viability of adaptive effector T cells appears to be necessary for the sustained remission of solid malignancies. Whether these adoptive cells were in the spleen, peripheral blood, or the tumor itself, our findings showed that the CD8^+^ T cells in the DC/tumor FC + LPS-RGD-Nb36-DOX group remained for almost 4 weeks as well as remained longer within tumor-bearing mice compared with control categories. The anticancer effect of sustained effector T cells is shown to be more enduring, and there is a persistent elevation of TNF-α as well as IFN-γ in mouse serum.

In conclusion, we have created a brand-new liposome loaded with doxorubicin and anti-CTLA-4 Nb that has been altered with RGD tripeptide. Our findings underline that liposomes can not only serve as a drug vector by modifying CTLA-4 inhibitors to block the CTLA-4/B7 signaling pathway upon CD8^+^ T cells, but also simultaneously load chemotherapy drugs, achieving an antitumor treatment strategy of chemotherapy combined with immunotherapy. Furthermore, we have offered preclinical knowledge regarding the LPS-RGD-Nb36-DOX possibility of therapy. When combined with LPS-RGD-Nb36-DOX, CD8^+^ T cells stimulated through the DC/tumor fusion vaccine could specifically destroy matched tumor cells, this seems to indicate a safe application, and its greater value is that this treatment strategy may reduce the dose of chemotherapeutic drugs and reduce the risk of adverse effects in antitumor therapy. This optimally designed co-loaded strategy could be helpful in improving effector cells growth, cell death, and even longevity in vivo thus lead to synergistic effect of chemotherapy combined with immunotherapy.

### Supplementary information

S table1

Supplementary figure legends

Supplementary Figure S1

Supplementary Figure S2

Supplementary Figure S3

Supplementary Figure S4

Supplementary Figure S5

Supplementary Figure S6

## Data Availability

The datasets generated and/or analyzed during the current study are available from the corresponding author upon reasonable request.
