# Supplementary material for: A novel doxorubicin/CTLA-4 blocker co-loaded drug delivery system improves efficacy and safety in antitumor therapy
Source: Cell Death Dis. 2024 Jun 1;15(6):386. doi: 10.1038/s41419-024-06776-6 (PMC11144200; doi:10.1038/s41419-024-06776-6)
Supplement: Supplementary file 1 — S table1 [file 41419_2024_6776_MOESM1_ESM.docx]

|  | Hydrodynamic size (nm) | PDI |
| --- | --- | --- |
| LPS | 112.45±0.80 | 0.197±0.003 |
| LPS-DOX | 113.64±2.45 | 0.201±0.006 |
| LPS-RGD-Nb36 | 111.10±3.36 | 0.209±0.004 |
| LPS-RGD-Nb36-DOX | 116.62±3.25 | 0.203±0.004 |

Table1. The hydrodynamic size and polydispersity index(PDI) of LPS-RGD-Nb36-DOX.
