# Supplementary material for: A novel doxorubicin/CTLA-4 blocker co-loaded drug delivery system improves efficacy and safety in antitumor therapy
Source: Cell Death Dis. 2024 Jun 1;15(6):386. doi: 10.1038/s41419-024-06776-6 (PMC11144200; doi:10.1038/s41419-024-06776-6)
Supplement: Supplementary file 2 — Supplementary figure legends [file 41419_2024_6776_MOESM2_ESM.docx]

**Supplementary Figure S1. The standard curve of DOX in LPS-RGD-Nb36-DOX synthesis.**

**Supplementary Figure S2.Generation and characterization of FC+****LPS-RGD-Nb36-DOX.** (A) TEM image for LPS-RGD-Nb36-DOX. (B) The Zeta Potential distribution of LPS-RGD-Nb36-DOX. (C) The size distribution of LPS-RGD-Nb36-DOX. (D) To test the CTLA-4 binding specificity of LPS-RGD-Nb36-DOX, flow cytometry was used to detect the expression of the His-tag in the CD8^+^ T cells that had been incubated with anti His-tag mAb. Meanwhile, these CD8^+^ T cells incubated with LPS-RGD-Nb36-DOX were again stained with anti CTLA-4 mAb, and their CTLA-4 expression was detected by flow cytometry.

**Supplementary Figure S3. Fusion of DCs and tumor cells.** (A) DCs and Hep3B, A-427 cells were co-cultured in the presence of PEG 2000, respectively. DCs were stained with PKH26 (red), tumor cells were stained with CFSE (green),and nuclei were stained with DAPI (blue). Scale bar= 50 μm. (B) DC/tumor fusion cells expressed high levels of costimulatory CD80, CD86 and MHC II molecules. *n*=3, ****P* < 0.001, *****P* < 0.0001.

**Supplementary Figure S4. Ex vivo fuorescence distribution of hearts, livers, spleens, lungs and kidneys. Fluorescence in major organs was quantifed using IVIS imaging software.**

**Supplementary Figure S5. Toxicity assessment for LPS-DOX.** (A)Representative images of hematoxylin-eosin staining in the primary organs of mice at the second day (100x , Scale bar=50 um). (B)Serum levels of ALT, AST, CK, CK-MB and LDH-L in the indicated treatment groups. *n*=3, ****P* < 0.001, *****P* < 0.0001, NS=*P* >0.05.

**Supplementary Figure S6.** **Construction of patient-derived HCC tumor xenograft mouse model.** (A) the collected HCC tissue was cut into 3 ~ 4 mm slices and inoculated subcutaneously into the left armpit for NOD/SCID mice. (B) Sections from tumor of PDX model were stained with hematoxylin-eosin and images were captured using fluorescence microscopy. Magnification, x400.
