# Supplementary figures and images for: A novel doxorubicin/CTLA-4 blocker co-loaded drug delivery system improves efficacy and safety in antitumor therapy
Source: Cell Death Dis. 2024 Jun 1;15(6):386. doi: 10.1038/s41419-024-06776-6 (PMC11144200; doi:10.1038/s41419-024-06776-6)

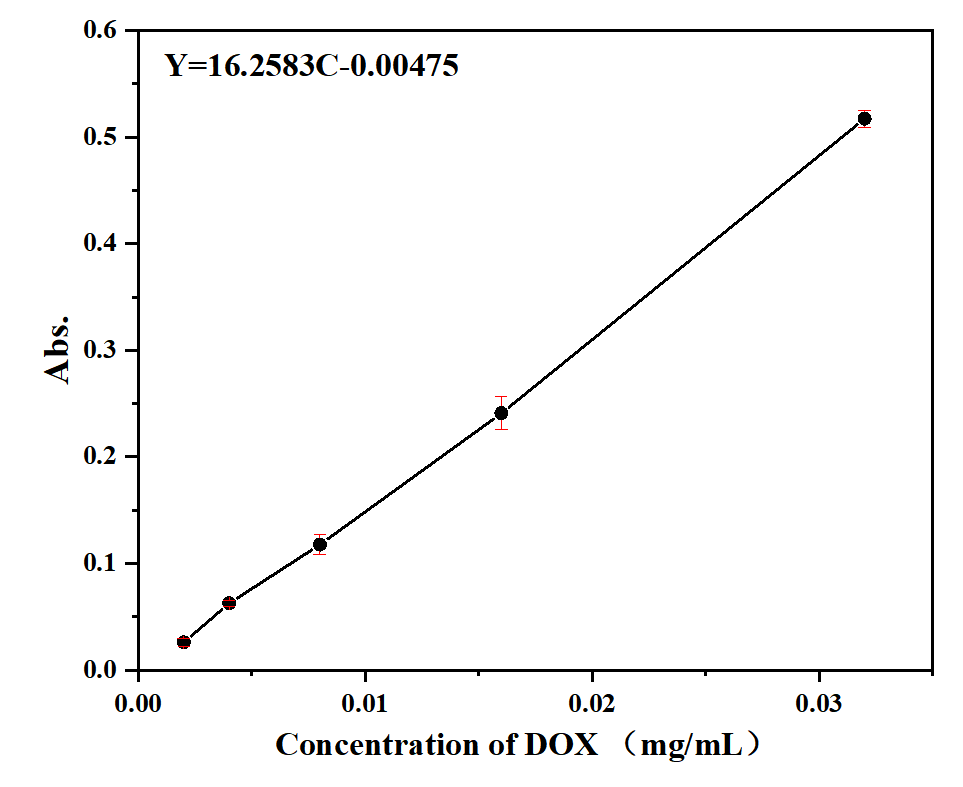

Supplement: Supplementary file 3 — Supplementary Figure S1 [file 41419_2024_6776_MOESM3_ESM.tif]

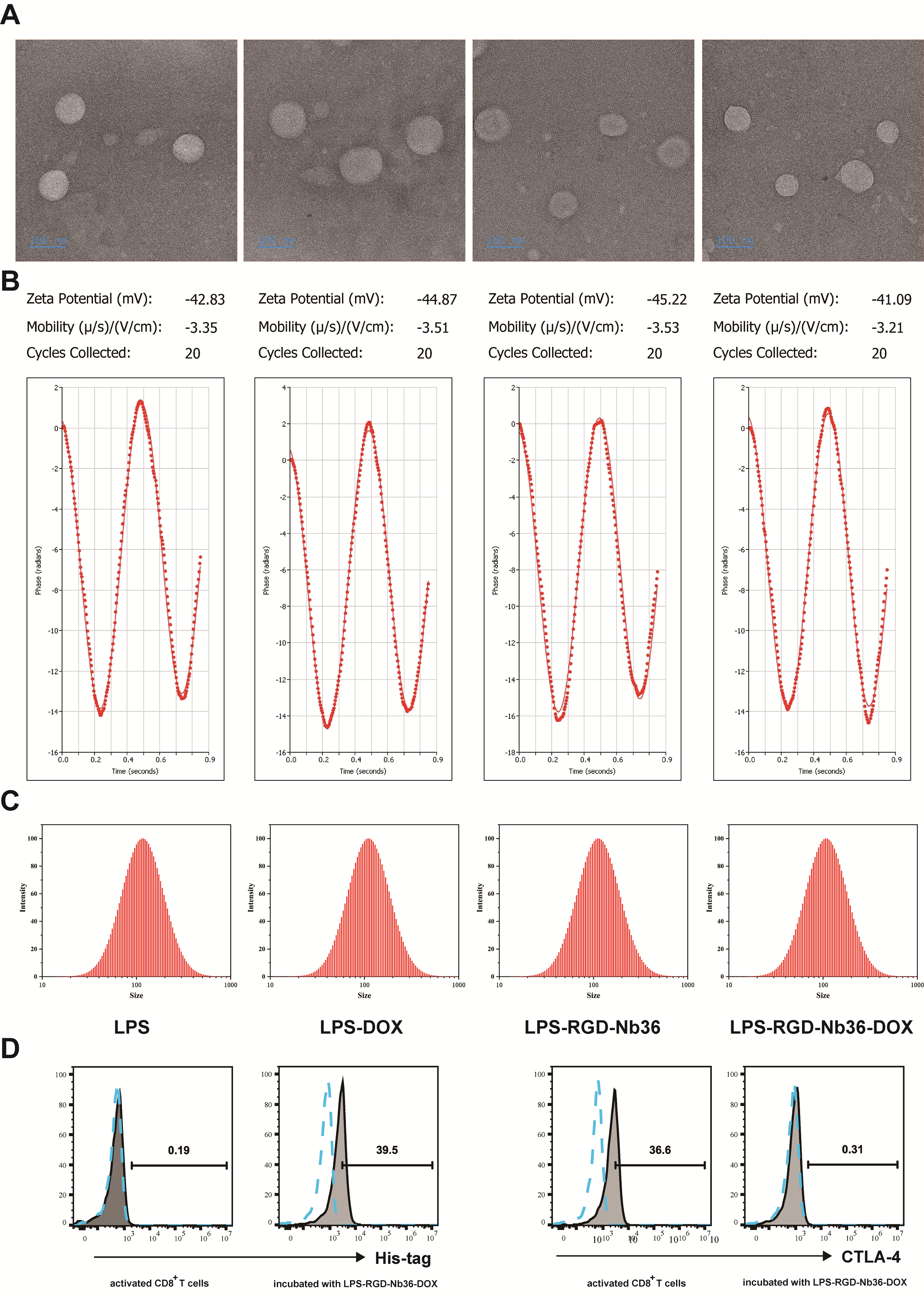

Supplement: Supplementary file 4 — Supplementary Figure S2 [file 41419_2024_6776_MOESM4_ESM.tif]

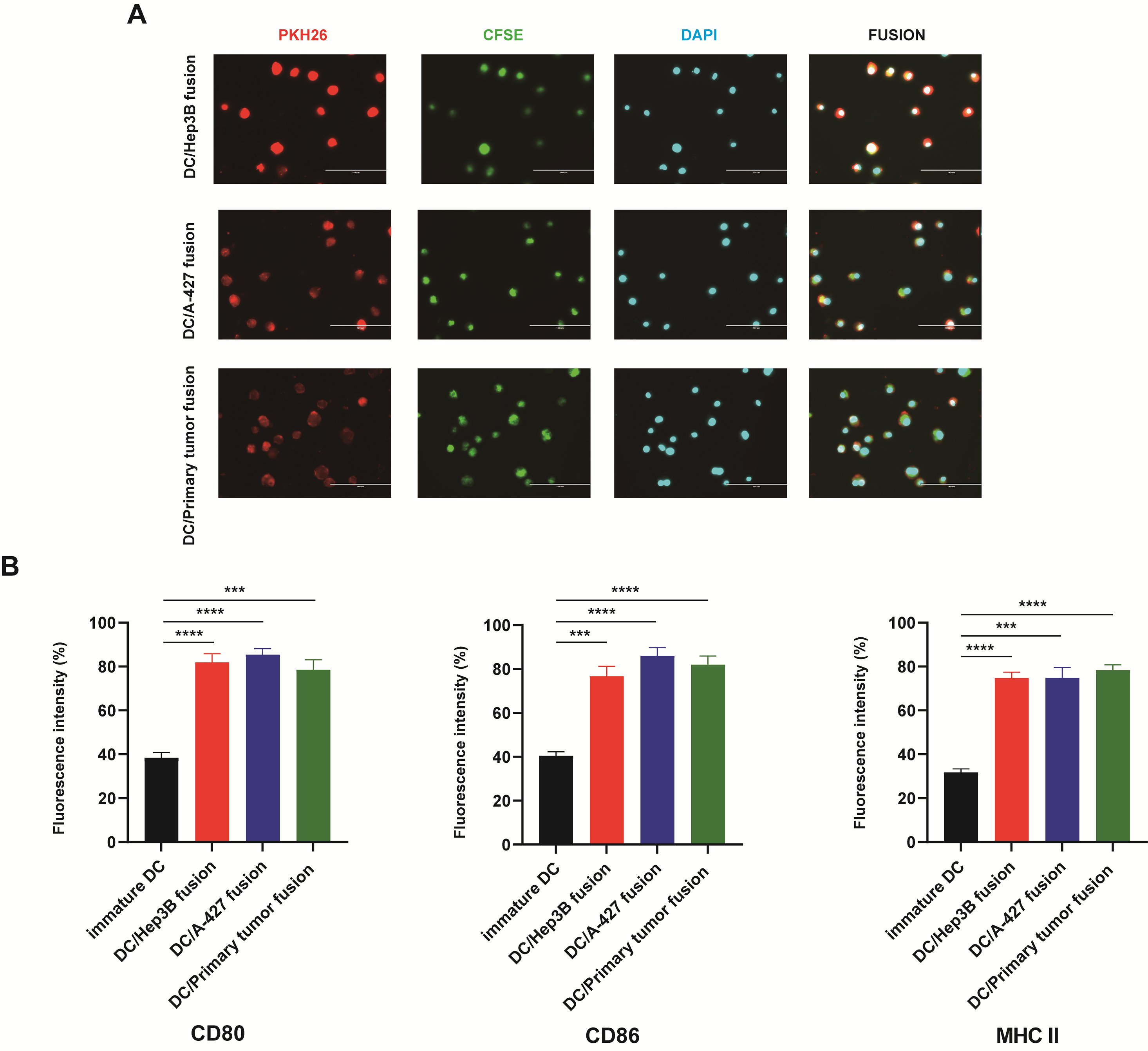

Supplement: Supplementary file 5 — Supplementary Figure S3 [file 41419_2024_6776_MOESM5_ESM.tif]

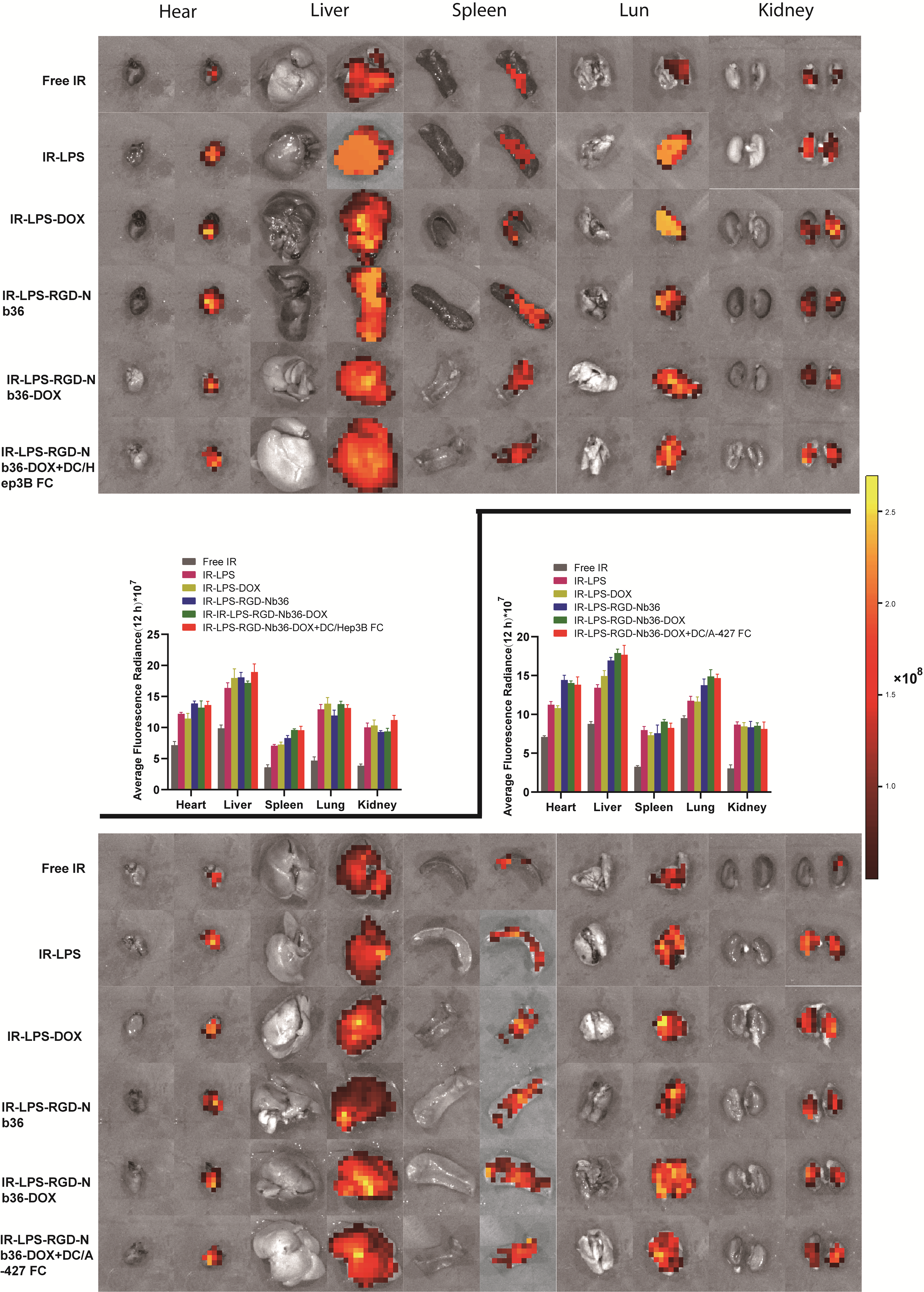

Supplement: Supplementary file 6 — Supplementary Figure S4 [file 41419_2024_6776_MOESM6_ESM.tif]

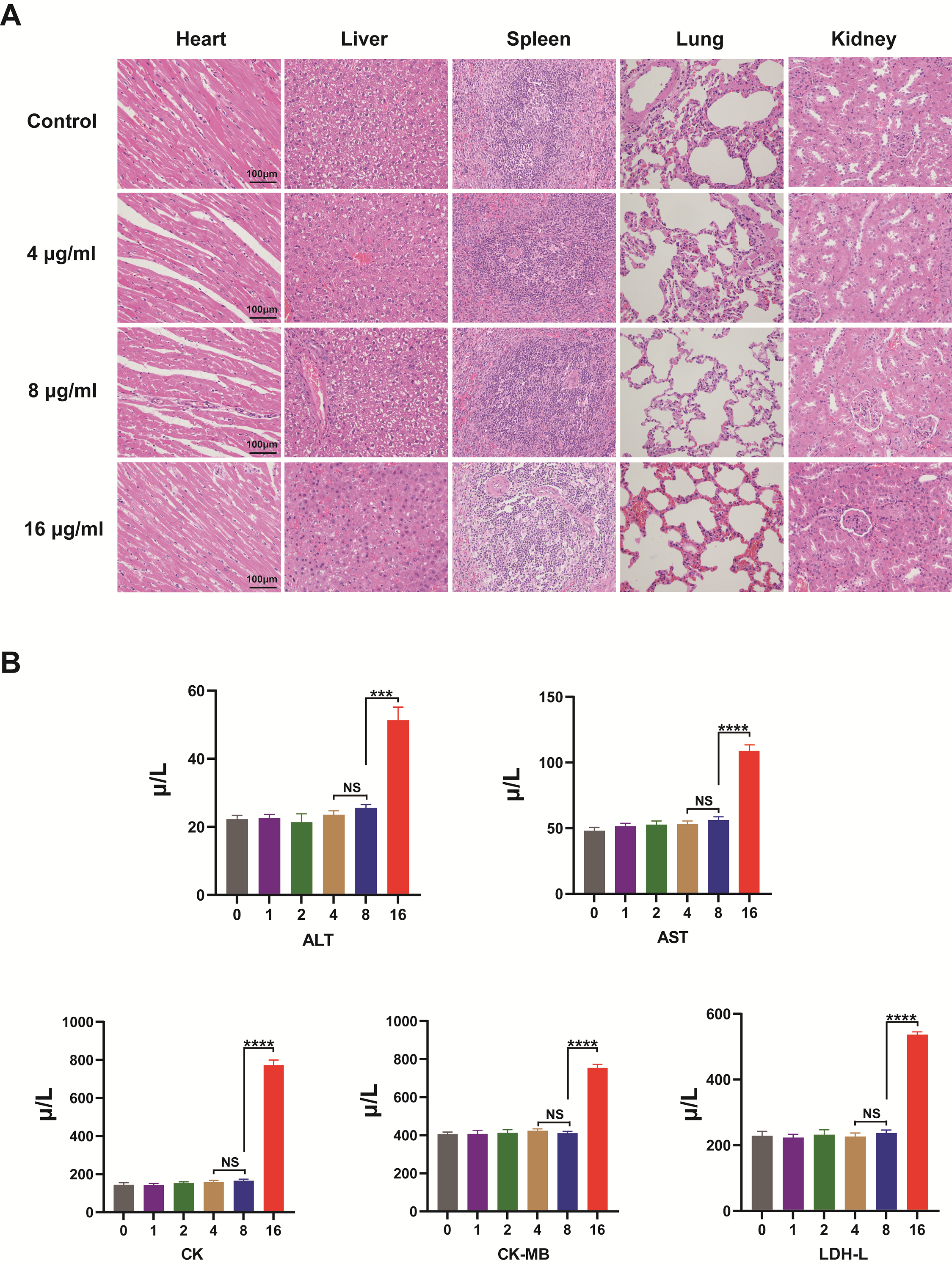

Supplement: Supplementary file 7 — Supplementary Figure S5 [file 41419_2024_6776_MOESM7_ESM.tif]

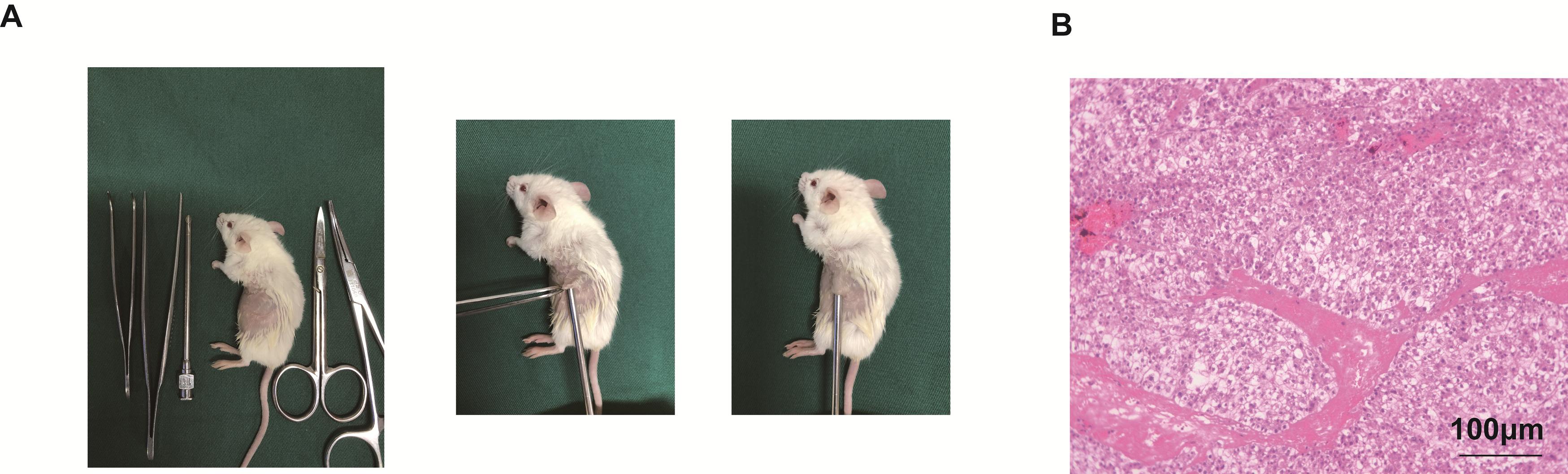

Supplement: Supplementary file 8 — Supplementary Figure S6 [file 41419_2024_6776_MOESM8_ESM.tif]
